# Vancomycin-Induced Neutropenia With Subsequent Perioperative Readministration: A Case Report

**DOI:** 10.7759/cureus.55858

**Published:** 2024-03-09

**Authors:** Yasutaka Shinoda, Teppei Kawabata, Kengo Ohashi, Eiseki Usami

**Affiliations:** 1 Pharmacy, Ogaki Municipal Hospital, Ogaki, JPN; 2 Neurosurgery, Ogaki Municipal Hospital, Ogaki, JPN

**Keywords:** ceftriaxone, readministration, thrombocytopenia, neutropenia, vancomycin

## Abstract

Vancomycin (VCM), an essential antibiotic for antimicrobial-resistant Gram-positive cocci, can lead to complications such as neutropenia. Here, we present a case of a 25-year-old male with noncommunicating hydrocephalus due to an intraventricular tumor who developed neutropenia during VCM therapy. Despite the suspected VCM-induced neutropenia, short-term readministration was deemed necessary for perioperative infection prophylaxis. This patient was readministered without neutropenia. A review of the literature revealed an earlier onset of VCM-induced neutropenia than that previously reported, emphasizing the importance of vigilant monitoring. Although readministration of VCM in patients with neutropenia is uncommon, it may be feasible with careful risk assessment, particularly in cases of mild neutropenia and short-term therapy. However, the mechanisms underlying VCM-induced neutropenia remain unclear, necessitating further research on the optimal management strategies.

## Introduction

Vancomycin (VCM) exhibits antibacterial activity against antimicrobial-resistant Gram-positive cocci, including methicillin-resistant *Staphylococcus aureus* [1]. Antimicrobial agents against these resistant organisms are limited, with VCM occupying an important position among them. Renal injury is a well-known adverse effect of VCM [2]. However, it rarely causes hematological abnormalities such as leukopenia [3,4]. VCM-induced neutropenia has been reported since around the 1960s, with an estimated incidence ranging from 2% to 12% [5-8]. Severe cases progressing to agranulocytosis have been documented, albeit more rarely [9]. Therefore, monitoring neutrophil count is recommended during VCM therapy [3]. Conversely, the clinical feasibility of readministering VCM in patients with a history of VCM-induced neutropenia remains unclear, likely due to the infrequency of such occurrences and subsequent rechallenges in clinical practice [7,10]. In this report, we described a case in which short-term readministration with VCM was deemed feasible in a patient with strong suspicion of VCM-induced neutropenia.

## Case presentation

Initial diagnosis and treatment

**Figure 1:**
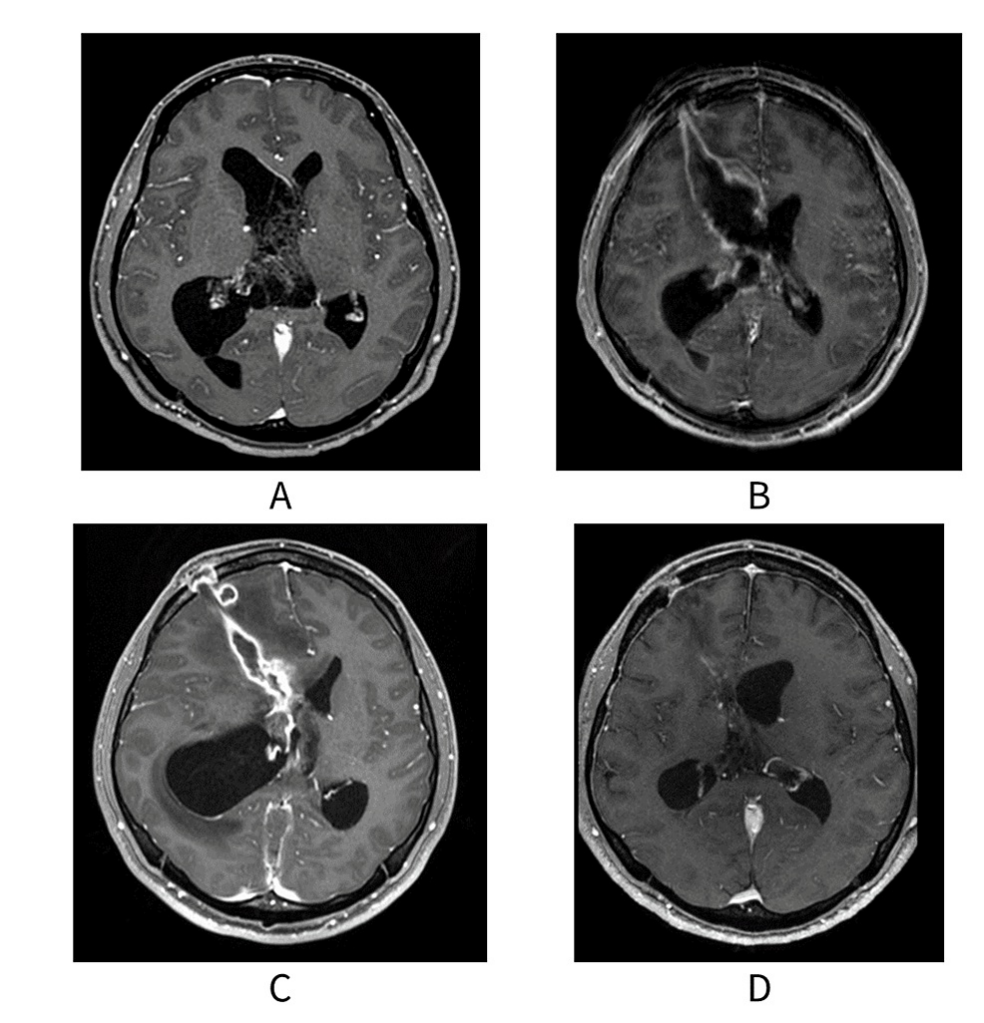
MRI images of this case are shown to illustrate the course of the condition. (A) Tumor within the ventricles of the brain (time of hospitalization). (B) Contrast enhancement was noted around the ventricular drain insertion site and surgical site, raising suspicion for postoperative meningitis. However, no obvious abscess was found (day eight). (C) Hydrocephalus was diagnosed following enlargement of the ventricles and progression of left hemiplegia (day 35). (D) Follow-up images after improvement of brain abscess. The abscess has disappeared and the enlargement of the ventricles has improved (day 171).

A 25-year-old male with no significant medical history presented to the emergency department with headache and vomiting. Patients had no abnormal liver, renal, or cardiac function. Nor was there any developmental disorder. Investigations revealed noncommunicating hydrocephalus due to an intraventricular tumor (Figure 1, panel A). The following day, the patient underwent ventriculostomy, followed by two endoscopic tumor resections, resulting in complete tumor removal. Pathological examination confirmed central neurocytoma. Postoperatively, oral lansoprazole (15 mg) was initiated in the patient as prophylaxis for peptic ulcers. After surgery, the patient progressed without disorientation or organ injury.

Complications

**Figure 2:**
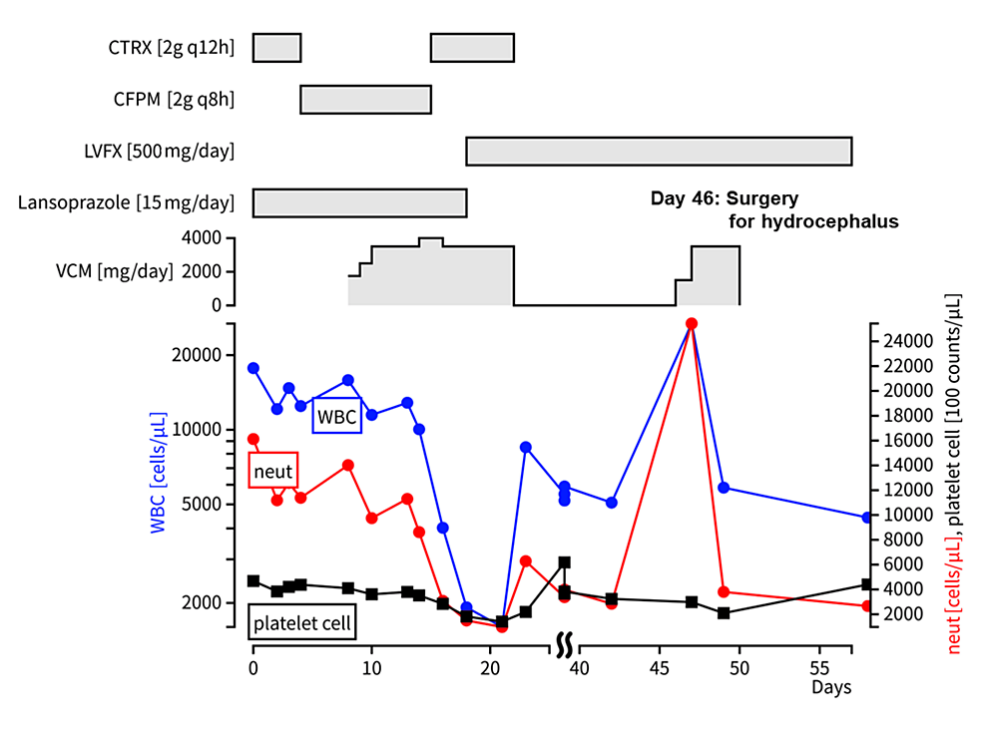
A timeline graph illustrating the drug usage and hematological changes in this case. CTRX: ceftriaxone; CFPM: cefepime; LVFX: levofloxacin; VCM: vancomycin; WBC: white blood cell count; neut: neutrophil count

On the sixth day postoperatively (day 0), sepsis was suspected due to persistent fever, and ceftriaxone (CTRX) 2 g was initiated every 12 h. The medication regimen and hematological parameters are summarized in Figure 2. On day four, *Pseudomonas aeruginosa* was isolated from the urine culture, leading to a change in antibiotic therapy to cefepime 2 g every 8 h. On day eight, contrast enhancement was noted on MRI around the ventricular drain insertion and surgical sites, raising suspicion of postoperative meningitis and necessitating the addition of VCM (Figure 1, panel B). The initial dose was 1750 mg followed by 1250 mg every 12 h. On day 10, owing to a VCM trough concentration of 5.6 mg/dL, the dose was increased to 1750 mg every 12 h. On day 11, the patient exhibited symptoms such as altered mental status, vomiting, and agitation. On day 14, with a VCM trough concentration of 8.9 mg/dL, the dose was further increased to 2000 mg every 12 h. As the patient continued to have disturbing symptoms, cefepime {CFPM}-induced encephalopathy was suspected, and the patient was switched from CFPM to CTRX on day 15. A brain abscess was suspected; therefore, perforator drainage was performed. On day 16, with a VCM trough concentration of 12.2 mg/dL, the dose was reduced to 1750 mg every 12 h. On day 18, the VCM trough concentration was calculated to be 10.2 mg/dL (area under the curve {AUC}: 501 μg×h/mL). *Mycoplasma hominis* was isolated from the surgical site, prompting the administration of oral levofloxacin (500 mg/day). At this point, mild neutropenia was noted with white blood cell and neutrophil counts of 1920 and 1500 cells/μL, respectively, leading to the discontinuation of lansoprazole. Fever (37-40°C) and slight impaired consciousness (Glasgow Coma Scale: 14) were present, but blood pressure and pulse were in the normal range. Respiratory, coagulation, hepatic, and renal functions were normal, and there was no evidence of sepsis. On day 21, the VCM serum concentration was 8.7 mg/dL, and the neutrophil count further decreased to 976 cells/μL, prompting cessation of VCM and CTRX. On day 23, a yeast-like fungus was isolated from the ventricular drain, leading to initiation of fosfluconazole. Neutrophil count had recovered to 6280 cells/μL without specific intervention. The next eight days were good and no paralysis was observed. However, around day 31, he began complaining of loss of appetite.

Perioperative period with readministration of VCM

On day 35, left hemiparesis developed, and a diagnosis of hydrocephalus was confirmed using contrast-enhanced MRI, necessitating emergency ventriculostomy. Fosfluconazole treatment was discontinued on day 36 (Figure 1, panel C). On day 46, because of worsening left hemiparesis, endoscopic ventriculostomy was performed. Methicillin-resistant coagulase-negative staphylococci were isolated from the removed ventricular drain on day 42, prompting the perioperative administration of VCM. The dose was 1500 mg on the day of surgery, followed by 1750 mg every 12 h. At this point, the neutrophil count was 2850 cells/μL. On day 49, the VCM trough concentration was 17.9 μg/mL (AUC: 720 μg×h/mL), and the treatment was discontinued. The neutrophil count was 3800 cells/μL, with no evidence of neutropenia. Postoperatively left hemiplegia improved. On day 57, levofloxacin therapy for *Mycoplasma hominis* brain abscess was completed, and the patient maintained a favorable clinical course (Figure 1, panel D).

## Discussion

VCM is a crucial antibiotic for resistant Gram-positive cocci such as methicillin-resistant *Staphylococcus aureus* and enterococci [1]. This is a valuable alternative for patients with beta-lactam antibiotic allergies [11]. Determining the appropriateness of VCM administration is clinically significant. In this case, although VCM-induced neutropenia due to VCM was strongly suspected, short-term readministration was feasible. The insights gained from this case are discussed below.

In this case, the VCM serum concentrations were generally within the therapeutic range [12]. However, neutropenia was confirmed between days 11 and 13 after the initiation of treatment. Neutropenia in this case was scored as 3 on the Naranjo scale, suggesting a "possible" association with VCM [13]. Black et al. reported that neutropenia due to VCM may depend more on prolonged administration than on the serum concentration or cumulative doses [3]. Additionally, Holz et al. reported that neutropenia due to VCM typically occurs 19-35 days after the initiation of therapy [4]. In this case, neutropenia occurred earlier than reported in these reviews, possibly because they mainly focused on severe neutropenia (absolute neutrophil count {ANC} <500 cells/μL). Vigilant monitoring of blood cell counts during VCM administration may lead to early detection of neutropenia, thus preventing its progression.

However, no consensus exists regarding the possibility of readministering VCM in cases of VCM-induced neutropenia, primarily because readministration is rarely performed in clinical practice [10]. Additionally, approximately half of patients who experienced neutropenia due to VCM developed neutropenia with teicoplanin. Therefore, it remains unclear whether teicoplanin is a suitable alternative [14]. In this case, the need for readministration of VCM was considered because of the requirement for perioperative infection prophylaxis after discontinuation of VCM due to neutropenia. Considering the mild nature of neutropenia, short duration of the intended administration, and lack of evidence for alternative perioperative antimicrobials other than VCM, readministration of VCM was deemed appropriate, resulting in no recurrence of neutropenia [15]. Schwartz reported that a single dose of VCM associated with coiling surgery for basilar artery aneurysms produced severe neutropenia (ANC: 6 cells/mm^2^) in a patient with VCM-induced neutropenia before complete recovery of blood cells [7]. Duff et al. reported a case of progression to agranulocytosis after VCM retreatment in a patient with thrombocytopenia after long-term VCM administration [16]. In contrast, no recurrence of neutropenia was observed upon readministration in this case. This could be attributed to the absence of neutropenia at the time of VCM readministration. Although this report is older, this highlights the importance of confirming neutrophil count recovery before readministration [17]. And in this case report, the administration period at the time of readministration was five days. Our case also had a short administration period of four days at the time of readministration. In addition, VCM-induced neutropenia progressed gradually in the present case. So, the risk of recurrence may be low with short-term readministration. In brief, this case suggests that VCM does not have to be given up in patients with previous VCM-induced neutropenia.

Various mechanisms have been proposed for VCM-induced neutropenia, with one prominent hypothesis involving the production of antineutrophil antibodies (including antineutrophil cytoplasmic antibodies {ANCA}) [9,18]. Indeed, case reports of severe neutropenia due to readministration have reported the presence of antineutrophil antibodies [7]. Therefore, it may be difficult to readministration when neutropenia occurs due to this mechanism. However, various mechanisms, including direct marrow suppression, inhibition of granulocyte production, and complement-mediated cell injury have been discussed, suggesting that readministration of VCM may be possible in some cases [18]. Immunological tests such as antineutrophil antibody testing were not performed in the present case; therefore, the mechanism of neutropenia remains unclear. Nevertheless, a modest reduction in platelet count was observed in this case, accompanied by neutropenia. So, it is more likely that some mechanism of VCM-induced hematopoietic dysfunction was involved, rather than a mechanism that acts directly on neutrophils, such as the production of antineutrophil antibodies. Future studies are needed to investigate the cause of VCM-induced neutropenia and its relevance to the risk of readministration.

In the present case, three points warrant consideration. First, CTRX can cause neutropenia. However, reports of neutropenia due to CTRX are scarce compared with those of VCM, and the onset is different (with past reports showing onset after more than two weeks in 10 of 12 cases) [19]. Therefore, the likelihood of adverse effects was considered to be low. Second, CFPM can cause neutropenia. Since antimicrobial-induced neutropenia generally recovers rapidly or gradually with discontinuation of antimicrobials, CFPM is unlikely to be the cause. In addition, according to a review of CFPM-induced neutropenia, the shortest time to onset of neutropenia was 16 days [20]. Based on the above, it is unlikely that this patient had CFPM-induced neutropenia. However, caution is necessary regarding the readministration of CTRX or CFPM similar drugs (beta-lactam antibiotics with a triazinone moiety on the R2 side chain). Third, neutropenia in the present case may have been caused by factors other than medications. However, this possibility is unlikely because there is no evidence to suggest sepsis. On the other hand, systemic inflammatory response syndrome or a compensated antiinflammatory syndrome is possible. However, considering the lack of vital sign changes other than fever and the complete absence of organ dysfunction, we consider this possibility unlikely. Since no reports of lansoprazole-induced neutropenia can be found at this time, it is most likely that the neutropenia seen in the present case was caused by VCM.

## Conclusions

Frequent hematological monitoring during VCM administration may help prevent severe neutropenia. Moreover, short-term readministration may be considered after an appropriate risk assessment in cases where VCM is discontinued owing to neutropenia. However, we cannot strictly exclude the possibility that VCM was not the reason for the neutropenia in the present case. Therefore, readministration to patients with a history of VCM-induced neutropenia should still be approached with caution. Also, the mechanisms underlying VCM-induced neutropenia remain unclear. Therefore, it is necessary to discuss the possibility of readministration along with detailed information such as the presence of antineutrophil antibodies and bone marrow biopsy findings.
